# Boredom in a Time of Uncertainty: State and Trait Boredom’s Associations with Psychological Health during COVID-19

**DOI:** 10.3390/bs12080298

**Published:** 2022-08-21

**Authors:** Emily R. Weiss, McWelling Todman, Emily Maple, Rebecca R. Bunn

**Affiliations:** Department of Psychology, The New School for Social Research, New York, NY 10011, USA

**Keywords:** COVID-19, pandemic, boredom, boredom proneness, state boredom, trait boredom

## Abstract

Throughout the COVID-19 pandemic, studies have demonstrated increases in boredom and its negative impact on mental health. This cross-sectional study examines state and trait boredom at four different points of the pandemic using an online sample of participants from the United States (*n* = 783). The results showed significant increases in boredom proneness, state boredom, substance use, loneliness, and distress. Boredom was associated with increases in each of these variables and a greater likelihood of testing positive for COVID-19. Moreover, the increases in distress, loneliness, and substance use became non-significant when controlling for boredom. Boredom proneness remained associated with all adverse outcomes when accounting for state boredom. In contrast, the relationships between state boredom and most adverse outcomes lost significance when controlling for boredom proneness, and state boredom was positively associated with increased hope for the future. Overall, the results suggest that high boredom proneness is an important vulnerability factor for poor psychological health and risky behaviors during the pandemic. However, high levels of recent state boredom, independent of boredom proneness, do not predict similarly negative outcomes. State boredom may indicate the extent to which one remains hopeful that circumstances will improve without resorting to risky, potentially maladaptive coping strategies.

## 1. Introduction

As the COVID-19 pandemic spread throughout the United States (US), individuals across the country remained locked inside their homes while businesses, schools, and places of recreation remained closed for weeks at a time. Such conditions are conducive to numerous psychological problems [1], including increased experiences of boredom. Over the past two years, studies have shown increases in state boredom during the COVID-19 pandemic throughout the world [2,3,4,5].

A large-scale longitudinal study of South Korean individuals demonstrated that boredom increased significantly over the first 11 weeks of the pandemic, and this effect was most pronounced for younger individuals. Notably, whereas many of the other study variables (e.g., wellbeing; life-satisfaction) showed periods of improvement before declining again, boredom increased linearly, showing no improvement throughout the study [2]. In studies conducted in France [3], Germany [4], and Ghana [5], participants were asked to provide retrospective ratings of their boredom levels before the pandemic and ratings of their current boredom levels. Similar to the findings from South Korea [2], the participants reported boredom increases following the pandemic’s onset [3,4,5].

At first glance, boredom may appear to be a relatively innocuous, albeit unpleasant, emotional state, and one that is to be expected given the lockdown conditions present in early-to-mid 2020. However, boredom is associated with numerous undesirable outcomes [6] and pathological behaviors. A large body of research has documented associations between high boredom at the state and trait levels and numerous pathological behaviors. Some examples include depression [7,8,9], eating disorders [10,11,12,13], substance abuse [14,15,16,17], and self-injury [12,18]. Notably, numerous studies worldwide have demonstrated the relationships between boredom and adverse mental health outcomes in the context of the COVID-19 pandemic [19,20,21,22,23,24,25].

In Austria, one study showed that individuals who reported increased substance use to cope during the pandemic also reported higher levels of boredom [19]. Similarly, in the US, young adults reported a significant increase in marijuana use due to boredom compared to pre-pandemic use [20]. These studies suggest that boredom plays an essential role in the increases in substance use observed across other studies conducted during the pandemic [1,26]. In terms of different mental and physical health outcomes, a study in China showed that reports of higher state boredom were related to increased sadness, depression, stress, and decreased perceptions of life as meaningful [21]. A separate study in China linked boredom proneness to several markers of emotional distress, including depression, anxiety, and fear [22]. In a sample of American college students, boredom mediated the association between reports of more severe loneliness and increased depressive symptoms [23]. Finally, multiple studies have linked boredom to slowed perceptions of time in the context of the pandemic [3,4], increased fear of the pandemic [24,25], and even a greater likelihood of contracting the virus [27].

The association between boredom and the increased likelihood of contracting COVID-19 may highlight another pandemic-related risk factor associated with high degrees of boredom. Specifically, higher levels of boredom might lead to greater difficulties complying with the often boredom-inducing lockdown and social distancing requirements [27,28]. Several studies have explored how individual differences in boredom vulnerability, or boredom proneness, affect compliance with COVID-19 safety protocols [27,28,29]. Across these studies, boredom-prone individuals were less likely to comply with COVID-19 protocols [27,28,29]. They were also more likely to perceive the pandemic as a hoax [27] and to find adherence to the protocols more difficult [28]. Additionally, a recent study demonstrated that boredom proneness continued to predict pandemic-related rule breaking a year after the pandemic’s onset [30].

A comprehensive review of these studies is beyond the scope of this article. However, these studies highlight boredom’s relevance in the wake of the COVID-19 pandemic and its effects on mental and physical health. In the present study, we aimed to provide an exploration of pandemic-related boredom from several unique perspectives.

First, we examined both state and trait boredom, allowing us to discern the unique contributions of each of these components of boredom. Many studies exploring pandemic-related boredom have focused on either boredom proneness, e.g., [22,27,28,29] or state boredom, e.g., [2]. Together, these studies suggest that highly boredom-prone individuals are at an increased risk for negative consequences associated with pandemic lockdowns [27,28,29], and that boredom rates have risen across many parts of the world, e.g., [2,3,4,5]. Thus, it is crucial to consider both state and trait forms of boredom when examining the impact of boredom on psychological outcomes amid COVID-19.

Although the subjective experience of boredom and boredom proneness are highly related constructs, they are, in fact, distinct [6]. State boredom, also called situation-dependent boredom, is thought to result from one’s current environment and is relatively transient. Specifically, once the conditions contributing to the boredom are removed or modified, the individual will experience reduced feelings of boredom [6]. Trait boredom, or boredom proneness, refers to an individual’s propensity to experience boredom. Boredom proneness varies amongst individuals, with some experiencing boredom more quickly and frequently than others [6,7].

It is expected that easily bored individuals (i.e., highly boredom-prone) are likely to be more frequently bored. However, it does not necessarily follow that all individuals who have been frequently bored in the recent past are also highly boredom-prone. The protracted lockdowns associated with the COVID-19 public health directives allowed us to explore this distinction. Specifically, we aimed to ascertain the extent to which adverse outcomes related to high boredom proneness depend on the co-occurrence of high levels of state boredom.

Moreover, previous data have suggested that state boredom and boredom proneness do not behave identically. For instance, state boredom has been associated with optimistic views of risk-taking [31], whereas boredom proneness has been associated with hopelessness [7]. Preliminary data from other studies have yielded similar findings [32], suggesting that boredom proneness and recent state boredom differentially affect how individuals perceive their futures. As the COVID-19 pandemic is characterized by uncertainty, the associations between boredom and perceptions of the future may be particularly relevant.

In the current study, we collected four waves of data to examine recent state boredom levels at four distinct points of the pandemic. Additionally, we could compare the pandemic recent state boredom levels with boredom rates obtained from a large US sample before the onset of the COVID-19 pandemic [33]. These existing data allowed us to directly compare pre-pandemic and mid-pandemic boredom levels using the same boredom measures in two large samples of individuals. To our knowledge, our study is one of the few studies conducted in the US that has not relied on participants’ recollections obtained during the pandemic to establish pre-pandemic levels of boredom [3,4,5]. Consistent with studies conducted in other parts of the world, e.g., [2], we expected that boredom rates would rise as the pandemic spread through the US and would show a significant increase compared to levels of recent state boredom before the pandemic.

We also aimed to examine relationships between boredom, psychological distress, wellbeing, and substance use in the context of the COVID-19 pandemic. Consistent with existing data, we expected that boredom would be positively associated with several markers of psychological distress (depression, anxiety, loneliness, and stress) and substance use. Additionally, we expected that boredom would be negatively associated with psychological wellbeing (optimism and hope). Finally, we aimed to determine whether state boredom and boredom proneness displayed unique relationships to these variables. Specifically, we examined each component of boredom’s (e.g., state or trait) associations with distress, wellbeing, and substance use while accounting for the other component.

## 2. Materials and Methods

### 2.1. Participants

This study consisted of a subset of data collected online using Amazon’s Mechanical Turk (M Turk). Across our four data collection time points, 783 individuals in the US participated in the study. Participants’ ages ranged from 18 to 73 (*M* = 37.53, *SD* = 11.81). Averaged across the four data collection time points, 56% of the sample identified as male, and 75% identified as white. See Table 1 for more detailed demographic information for each data collection point. Pre-pandemic comparison data (*n* = 1006) were collected in 2018 from Amazon’s M Turk. In this sample, 62% identified as female, 76% identified as white, and ages ranged from 18 to 87 (*M* = 35.80, *SD* = 12.51) [33].

### 2.2. Measures

#### 2.2.1. Boredom Proneness

The Short Boredom Proneness Scale (SBPS) [34], a shortened version of the original 28-item Boredom Proneness Scale [7], was used to assess individuals’ general propensity to experience boredom. Participants rate the degree to which they agree or disagree with eight statements assessing their tendency to become bored. Participants rate the items on a scale of 1–7. The SBPS includes statements such as, “I often find myself at ‘loose ends’”, not knowing what to do”. The SBPS has been validated and shows good convergent validity with constructs associated with boredom proneness [34]. The SBPS showed excellent internal consistency in the present study, Cronbach’s α = 0.93.

#### 2.2.2. Recent/Frequent Boredom Experiences

The State Boredom Measure (SBM) [9] was used to assess experiences of recent boredom in terms of attributions, frequency, tolerance, and intensity over the past two weeks. The SBM shows good convergent validity with other boredom measures and constructs associated with boredom [9]. Participants rate each of the eight items on a 7-point Likert-type scale. Items include statements such as, “over the last two weeks: How often would say that you can remember feeling bored?” The original conceptualization of the SBM did not include a formal summary score [9]. More recently, however, given its good internal consistency (Cronbach’s α = 0.81) and the high convergent validity with other boredom measures, it has become common practice to compute a summary score by adding the scores from the eight items [9]. A summary score was used in the present study, and the SBM showed excellent internal consistency, Cronbach’s α = 0.88.

#### 2.2.3. Psychological Distress

The Depression, Anxiety, and Stress Scales (DASS) [35] were used to measure psychological distress. In this study, we used the 21-item version of the scale. Participants answered questions regarding their stress, anxiety, and depressive symptoms over the previous week. Participants rate all items on a scale of 0–3. Each subscale displayed excellent internal consistency: Cronbach’s α = 0.91 for stress, α = 0.93 for depression, and α = 0.93 for anxiety. The DASS summary score also displayed excellent reliability, Cronbach’s α = 0.97.

#### 2.2.4. Loneliness

The Revised UCLA Loneliness Scale (UCLA-LS) [36] was used to assess loneliness. This 20-item measure consists of statements such as, “there is no one I can turn to”. Participants rate all items on a 4-point scale. The UCLA-LS showed excellent internal consistency in the present study, Cronbach’s α = 0.89.

#### 2.2.5. Substance Use

Participants rated how frequently they used alcohol, marijuana, or other types of drugs (Never–Daily). Additionally, they rated the level of change in their alcohol, marijuana, and other drug use since the pandemic’s beginning (Much less frequent use–Much more frequent use).

#### 2.2.6. Optimism

Participants completed the Revised Life Orientation Test (LOT-R) [37] as a measure of optimism. This scale contains ten items, four of which are filler items dropped before data analysis. The remaining six items include statements such as “Overall, I expect more good things to happen to me than bad.” Participants rate each item on a scale of 0–4. The LOT-R showed acceptable internal consistency in the present study, Cronbach’s α = 0.79.

#### 2.2.7. Hopefulness

Participants completed two measures of hope. They completed the Hope Scale (HS) [38], which consists of 12 items, four of which are filler items that are dropped before data analysis. The remaining eight items are rated on a scale of 1–4 and include statements such as “I meet the goals I set for myself”. The HS can be divided into two subscales, Agency and Pathways. However, in the present study, a summary score was used. It showed good internal consistency, Cronbach’s α = 0.82. Participants also completed a 10-item measure assessing their appraisals of their lives three years in the future. This measure asked participants to rate on a scale of 1–7 the degree to which they agree or disagree with statements such as, “I will have chances to do a lot of things I enjoy”. This measure will be called the Future Appraisals Scale (FAS) for this study. It showed excellent internal consistency in the present study, Cronbach’s α = 0.92.

#### 2.2.8. COVID-19 Questions

Participants rated their fear of COVID-19 infection and whether or not they had tested positive for COVID-19. In the third and fourth waves of data collection, we asked participants to list the COVID precautions (if any) to which they were adhering (*n* = 383). These included standard recommendations such as handwashing, social distancing, and avoiding activities outside the home. In the complete survey, we asked participants additional questions about their feelings about the pandemic. However, these will be reported elsewhere.

### 2.3. Procedure

This study was approved by the Institutional Review Board of The New School for Social Research (Initial: #2020-45, 30 March 2020; Mod 1: #2020-45, 30 May 2020). Data were collected from unique respondents at four time points, occurring from March to July 2020 (total *n* = 783). At each time point, the survey was available for one week. Specifically, the data collection time points occurred from 31 March to 8 April 2020 (T1), 30 April to 8 May 2020 (T2), 30 May to 8 June 2020 (T3), and 30 June to 9 July 2020 (T4). We excluded participants who completed the survey at one time point from participating in future time points to ensure that each sample was unique. All participants provided electronic informed consent before beginning the study and were given contact information for a Disaster Distress Hotline in case they experienced distress related to the study’s content. All individuals who completed the study were compensated USD 1.00 for their participation. To improve data quality, we utilized a brief attentional filter at the beginning of the study. Overall, we excluded 213 participants for responding incorrectly to the attention check.

#### Statistical Analyses

Data were analyzed using IBM’s SPSS, Version 28 for Mac (IBM Corp, Armonk, NY, USA, accessed on 19 August 2022). Pearson’s bivariate correlations and partial correlations were used to examine associations between continuous variables. One-way analyses of variance (ANOVA) and one-way analyses of covariance (ANCOVA) were used to examine differences between the four time points. The differences between pre- and mid-pandemic recent state boredom scores were examined using an independent sample *t*-test.

## 3. Results

### 3.1. Demographic Differences

Previous studies have associated younger age and male gender with higher boredom proneness [39]. Thus, we examined differences in these two demographic variables between the four time points. There were no significant differences in the proportion of males and females included in each time point *χ*^2^(3) = 2.01, *p* = 0.570. However, a one-way analysis of variance (ANOVA) revealed a significant difference in age across the four time points, *F*(3, 1225.50) = 9.29, *p* < 0.001 η*p*^2^ = 0.04. Participants included in T4 were significantly younger than those included in T1 (*M_diff_* = −5.84, *SE* = 1.17, *p* < 0.001, 95% CI [−8.91, −2.77], and T3 (*M_diff_* = −4.47, *SE* = 1.19, *p* = 0.001, 95% CI [−7.61, −1.28]. None of the other time points differed from one another. Due to the differences in age across the time points, age was used as a covariate when examining boredom differences among the various data collection points.

Compared to the data collected before the pandemic, individuals in the present sample were significantly older *t*(1784) = 2.96, *p* = 0.003, *M_diff_* = 1.73, *SE* = 0.58, 95% CI [0.58, 2.87]. There was also a significantly higher proportion of female respondents in the pre-pandemic sample, *χ*^2^(1) = 59.81, *p* < 0.001. Thus, age and gender were used as covariates when comparing pre-pandemic data to the data collected in the present study.

### 3.2. Changes in Boredom Levels

**Figure 1:**
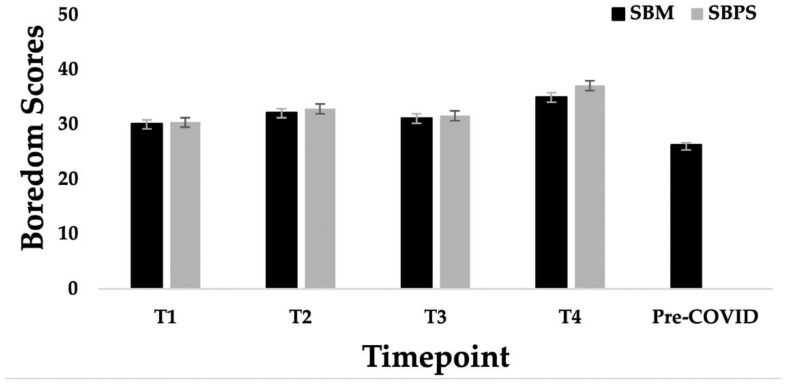
Differences in state boredom and boredom proneness across time points. Error bars represent standard error; T1 = time point 1; T2 = time point 2; T3 = Time point 3; T4 = Time point 4; SBM = State Boredom Measure; SBPS = Short Boredom Proneness Scale.

A one-way ANOVA demonstrated that reports of recent state boredom (*F*(3, 779) = 9.20, *p* < 0.001, η*p*^2^ = 0.03) increased from March to July. Pairwise comparisons demonstrated that participants reported significantly higher rates of recent state boredom at T4 (*M* = 35.08, *SE* = 0.69, 95% CI [33.72, 36.44]) compared to all other time points. Notably, these results became non-significant when controlling for boredom proneness and age, *F*(3, 774) = 0.21, *p* = 0.892, η*p*^2^ = 0.001. Interestingly, boredom proneness also increased from March to July (*F*(3, 779) = 13.23, *p* < 0.001, η*p*^2^ = 0.05) and followed the same pattern as recent state boredom. Specifically, participants at T4 reported higher rates of boredom proneness (*M* = 37.17, *SE* = 0.80, 95% CI [35.60, 38.74]) compared to all other time points. However, when controlling for recent state boredom and age, the difference remained significant, *F*(3, 774) = 3.18, *p* = 0.023, η*p*^2^ = 0.01, with participants reporting higher boredom proneness at T4 than at T1. The means for boredom proneness and recent state boredom at each time point are displayed in Table 2, and the differences between time points are displayed in Figure 1.

Significant differences in recent state boredom reports were also observed when compared to pre-pandemic recent state boredom scores, *t*(1,787) = 12.29, *p* < 0.001, *d* = 0.59. On average, study participants reported significantly lower rates of recent state boredom before the pandemic (*M* = 26.36, *SD* = 9.94) than during the pandemic (*M* = 32.19, *SD* = 9.98). The differences in recent state boredom before and during the pandemic are displayed in Figure 1. Notably, controlling for age and gender did not alter these results, *F*(3, 1778) = 146.21, *p* < 0.001, η*p*^2^ = 0.08.

### 3.3. Boredom and COVID-19

Across T3 and T4, participants who reported higher rates of boredom proneness also endorsed taking a fewer number of COVID-19 precautions, *r*(370) = −0.19, *p* < 0.001. Of note, fourteen participants were excluded from this analysis for providing invalid responses (i.e., selecting “none” and one or more COVID-19 precaution). The exclusion of these participants did not alter the results. This relationship remained significant when controlling for recent state boredom *r*(368) = 0.25, *p* < 0.001. Conversely, recent state boredom was not associated with COVID-19 precautions, *r*(370) = −0.02, *p* = 0.676). However, when controlling for boredom proneness, individuals with more recent and frequent state boredom reported taking more COVID-19 precautions, *r*(368) = 0.17, *p* < 0.001.

In terms of COVID-19 infection rates, individuals who reported that they had tested positive for COVID-19 also reported higher levels of boredom proneness (*M* = 40.33, *SD* = 8.85) compared to individuals who had not tested positive (*M* = 31.29, *SD* = 11.47), *t*(301.947) = 10.78, *p* < 0.001, *d* = 0.82. Controlling for recent state boredom did not alter these results, *F*(2, 780) = 52.91, *p* < 0.001, η*p*^2^ = 0.06. Individuals who tested positive for COVID-19 also reported higher rates of recent state boredom (*M* = 36.14, *SD* = 9.20) than those who had not tested positive (*M* = 31.20, *SD* = 9.92), *t*(781) = 5.65, *p* < 0.001, *d* = 0.51. However, rates of recent boredom did not differ significantly after controlling for boredom proneness, *F*(2, 780) = 2.10, *p* = 0.148, η*p*^2^ = 0.003.

Finally, individuals who reported more concern about COVID-19 reported higher levels of both boredom proneness (*r*(782) = 0.22, *p* < 0.001) and recent state boredom (*r*(782) = 0.28, *p* < 0.001. However, recent state boredom remained associated with pandemic-related concerns when controlling for boredom proneness (*r*(780) = 0.18, *p* < 0.001), whereas the relationship between boredom proneness and COVID-19 concerns was no longer significant after accounting for recent state boredom (*r*(780) = 0.02, *p* = 0.657).

### 3.4. Boredom and Psychological Health

The DASS summary score was used to examine changes in distress over the four time points and showed significant increases in depression, anxiety and stress scores, *F*(3, 779) = 12.15, *p* < 0.001, η*p*^2^ = 0.05. Specifically, distress reported at T4 was significantly higher than distress reported at all other time points (*M_diff’s_* 5.25–9.17, *p*’s < 0.05). Controlling for age did not alter these results, *F*(3, 775) = 9.08, *p* < 0.001, η*p*^2^ = 0.03. However, when additionally controlling for boredom proneness and recent state boredom, the increases in psychological distress became non-significant, *F*(3, 773) = 1.80, *p* = 0.145, η*p*^2^ = 0.007.

Similarly, loneliness also increased over the four time points, *F*(3, 779) = 7.91, *p* < 0.001, η*p*^2^ = 0.03. Specifically, individuals reported significantly lower loneliness at T1 compared to reports at T2 (*M_diff_* = −2.86, *SE* = 1.03, *p* = 0.034, 95% CI [−5.59, −0.13]), and T4 (*M_diff_* = −4.45, *SE* = 1.03, *p* < 0.001, 95% CI [−7.17, −1.74]). Individuals also reported significantly more loneliness at T4 compared to T3 (*M_diff_* = 3.84, *SE* = 1.05, *p* = 0.002, 95% CI [1.07, 6.62]). When controlling for age, the differences between T1 and T2 became marginally significant. Additionally, when controlling for recent state boredom and boredom proneness, the differences observed in loneliness scores became non-significant, *F*(3, 773) = 1.82, *p* = 0.143, η*p*^2^ = 0.007.

Finally, optimism decreased significantly over the four time points, *F*(3, 779) = 3.36, *p* = 0.018, with individuals at T4 reporting lower optimism than individuals at T1 (*M_diff_* = −1.12, *SE* = 0.41, *p* = 0.04, 95% CI [−2.21, −0.03]), and T3 [*M_diff_* = −1.16, *SE* = 0.42, *p* = 0.038, 95% CI [−2.23, −0.04]]. However, when controlling for age, the difference across time points became non-significant, *F*(3, 775) = 1.91, *p* = 0.136, η*p*^2^ = 0.007. Hope and appraisals of the future did not differ across the four time points.

Correspondingly, Pearson’s bivariate correlations demonstrated that both boredom proneness and recent state boredom were positively associated with depression, anxiety, stress, and loneliness and negatively associated with optimism (*r*’s 0.24–0.70, *p*’s < 0.001). Hope and appraisals of the future were unassociated with boredom proneness but showed modest positive correlations with recent state boredom (*r*’s 0.08–0.12, *p*’s < 0.05). These relationships are displayed in Table 3.

When controlling for recent state boredom, boredom proneness remained associated with higher reports on all psychological distress variables. Specifically, boredom proneness continued to show moderate to large positive associations with loneliness (*r*(780) = 0.44, *p* < 0.001), depression (*r*(780) = 0.55, *p* < 0.001), stress (*r*(780) = 0.52, *p* < 0.001), and anxiety (*r*(780) = 0.53, *p* < 0.001). Additionally, boredom proneness showed small to moderate negative associations with levels of optimism (*r*(780) = 0.34, *p* < 0.001), hope (*r*(780) = −0.15, *p* < 0.001), positive appraisals of the future (*r*(780) = −0.15, *p* < 0.001).

When accounting for boredom proneness, a distinct pattern emerged between recent state boredom and the psychological health variables. Specifically, a small positive relationship was observed between recent state boredom, hopefulness (*r*(780) = 0.17, *p* < 0.001), and more positive appraisals of the future (*r*(780) = 0.19, *p* < 0.001). Conversely, recent state boredom was found to be no longer associated with depression, stress, or anxiety, with correlation coefficients ranging from 0.004–0.04. Finally, recent boredom showed a modest positive association with optimism (*r*(780) = 0.09, *p* = 0.009), and a modest negative association with loneliness (*r*(780) = 0.08, *p* = 0.017).

### 3.5. Boredom and Substance Use

Mean rates of recreational drug use (*F*(3, 779) = 7.13, *p* < 0.001, η*p*^2^ = 0.03) and marijuana use (*F*(3, 779), = 6.47, *p* < 0.001, η*p*^2^ = 0.02) increased significantly from March to July.

Specifically, mean rates of marijuana use were higher at T4 than at T1 (*M_diff_* = 0.40, *SE* = 0.13, *p* = 0.009, 95% CI [0.07, 0.74]), and T3 (*M_diff_* = 0.54, *SE* = 0.13, *p* < 0.001, 95% CI [0.19, 0.88]). The differences between T1 and T4 became marginally significant when controlling for age. The same pattern was observed for other drug use; usage rates were higher at T4 than at T1 (*M_diff_* = 0.45, *SE* = 0.12, *p* < 0.001, 95% CI [0.13, 0.77]), and T3 (*M_diff_* = 0.52, *SE* = 0.12, *p* < 0.001, 95% CI [0.19, 0.85]). Controlling for age did not alter these results. Mean rates of alcohol use did not significantly differ across the time points, nor did self-reported changes in substance use frequency. Finally, when controlling for boredom proneness and recent boredom in addition to age, the increase in the mean rates of drug (*F*(3, 773) = 1.26, *p* = 0.288, η*p*^2^ = 0.005) and marijuana use (*F*(3, 773) = 2.09, *p* = 0.10, η*p*^2^ = 0.008) from March to July became non-significant.

Levels of recent state boredom were positively associated with mean rates of alcohol, drug, and marijuana use (*r*’s = 0.32–42, *p*’s < 0.001) and with self-reported increases in the use of each of these substances (*r*’s = 0.18–0.21, *p*’s < 0.001). Similarly, boredom proneness was associated with mean rates of alcohol, drug, and marijuana use (*r*’s = 0.33–0.56, *p*’s < 0.001) and self-reported increases in the use of each of these substances (*r*’s = 0.18–0.23, *p*’s < 0.001). These relationships are displayed in Table 4.

When controlling for recent state boredom, boredom proneness remained positively associated with mean rates of marijuana (*r*(780) = 0.31, *p* < 0.001), drug (*r*(780) = 0.23, *p* = 0.001), and alcohol use (*r*(780) = 0.15, *p* < 0.001). Participants endorsing higher levels of boredom proneness also reported greater changes in their drug use (*r*(780) = 0.13, *p* < 0.001) and boredom proneness was modestly associated with reports of changes in alcohol use (*r*(780 = 0.08, *p* = 0.025). Conversely, when controlling for boredom proneness, recent state boredom remained positively associated with mean rates of alcohol use (*r*(780) = 0.12, *p* < 0.001), but no other substances. Finally, recent state boredom continued to show modest associations with self-reported changes in alcohol (*r*(780) = 0.08, *p* < 0.019) and marijuana use (*r*(780) = 0.07, *p* = 0.042).

## 4. Discussion

This study examined boredom proneness and recent boredom experiences across the first several months of the COVID-19 pandemic. Consistent with previous research from around the world [2,3,4,5], participants reported higher rates of boredom towards the middle of the pandemic compared to the beginning stages. Additionally, recent state boredom scores were significantly higher compared to a large sample of participants surveyed before the onset of COVID-19. Additionally, congruent with previous studies, individuals with higher rates of boredom proneness reported worse outcomes in terms of mental health, substance use, hope for the future, and the extent to which they were taking precautions against COVID-19. Notably, they were also more likely to have tested positive for the virus, which might relate to the fewer pandemic precautions they endorsed.

Another notable finding from this study was the observed increase in psychological distress, loneliness, and substance abuse across the four time points tested. Moreover, these differences became non-significant after accounting for levels of recent state boredom and boredom proneness. Thus, boredom levels may be an essential controlling variable in individuals’ decreases in mental health and increases in substance use during the early months of the pandemic.

A unique aspect of this study was that it examined the contributions of boredom proneness and recent state boredom, together and separately. The findings demonstrated that boredom proneness is strongly associated with adverse outcomes, even in the absence of more frequent state boredom. Specifically, higher boredom proneness remained associated with stress, anxiety, depression, substance use, and a bleaker perception of the future. It also remained associated with a higher likelihood of contracting the virus and taking fewer precautions.

Interestingly, we observed a distinct pattern when examining the impact of recent state boredom on these same outcomes. In the absence of boredom proneness, high recent state boredom levels were no longer positively associated with depression, anxiety, stress, or loneliness. Although they were associated with more frequent alcohol use, and modestly associated with self-reported changes in marijuana use, recent state boredom levels were no longer associated with higher overall marijuana use or other types of recreational drug use. When controlling for boredom proneness, recent state boredom was no longer related to a greater likelihood of contracting COVID-19 and was positively associated with taking a greater number of precautions against the virus. It is possible that, whereas boredom proneness puts individuals at a greater risk of breaking COVID-19 protocols [26,27,30], state boredom does not pose the same risk on its own.

In the absence of boredom proneness, individuals experiencing more frequent boredom may find more adaptive, safer ways of managing this unpleasant emotion. Another finding from this study showed that after controlling for boredom proneness, recent state boredom was no longer related to a bleaker view of the future. In fact, individuals reporting higher rates of recent state boredom endorsed higher hopes for the future and more positive appraisals of what their lives would look like in several years.

The association between recent experiences of boredom and hope is consistent with previous data collected in our lab [32] that has shown a positive relationship between self-reported recent state boredom levels and hopeful perceptions of the future. It is also consistent with a recent study demonstrating that experimentally induced state boredom can result in more optimistic perceptions of risk [31]. Although seemingly counterintuitive, we believe this finding is entirely consonant with the presumed evolutionary function of boredom. Indeed, as a putative signal for the need to invest one’s attentional resources differently when current investment strategies are failing to yield satisfactory returns [6,40,41], it seems reasonable to assume that feelings of boredom must also include the implicit belief and expectation that opportunities for better investments remain possible, if not probable. If this conjecture is correct, then it is possible to conceive of boredom proneness partly as a reflection of the individual’s capacity to manage their attentional investments effectively without resorting to high-risk strategies. In other words, highly boredom-prone individuals may be distinguished by their willingness to embrace the proposition that better investments are unlikely to materialize unless relatively high-risk and potentially maladaptive coping strategies are adopted. Such an approach to boredom management would effectively reduce the incidence and severity of subjective boredom. Still, as a downside, it would also increase the incidence and severity of adverse outcomes. Such a dynamic would explain why, in the absence of boredom proneness, state boredom was no longer related to many adverse mental health outcomes in this study.

Another notable finding from this study was that individuals reporting higher rates of boredom also reported more concerns about contracting COVID-19. Boredom’s association with greater concerns about the virus replicates studies from Italy [24] and Germany [23], showing that higher rates of state boredom were associated with greater fear of the coronavirus. Interestingly, when controlling for recent state boredom levels in the present study, the relationship between boredom proneness and COVID-19 concerns became non-significant. This finding supports the idea that recent state boredom levels and boredom proneness may not show identical relationships with health-related behaviors and outcomes. This finding is also consistent with previous results and those of the present study, which demonstrate that boredom proneness is associated with fewer COVID-19 precautions, more difficulty following pandemic protocols [27,28,29,30], and beliefs that COVID-19 is a hoax [27]. Conversely, the positive association between high levels of recent state boredom and fears of the virus is consistent with the greater number of COVID-19 precautions endorsed by highly bored individuals in the present study. Additionally, if highly bored individuals are more concerned about the virus, they may be more inclined to find ways to cope safely and effectively. This might relate to the higher rates of hopefulness observed by highly bored individuals in the present study.

Finally, it is worth noting that boredom proneness scores increased across the time points tested in this study, despite its characterization as a trait variable. As the sample consisted of unique respondents at each time point, individual differences may explain this increase. However, the samples were collected from the same data collection source (M Turk) and did not differ demographically in the proportion of male and female respondents. Although the samples did differ in age, the difference in boredom proneness scores remained significant even when accounting for age. Moreover, the large sample size at each time point (*n* = ~200) makes it less likely that this difference is explainable by individual differences alone. Conversely, preliminary evidence suggests that boredom proneness scores can increase when individuals are exposed to boring environments [42]. It is possible that the prolonged boring environment associated with lockdowns increased boredom proneness scores as the pandemic continued to spread.

### Limitations and Directions for Future Research

Although this study provided a broad survey of boredom in the context of COVID-19, there are several notable limitations. The online data collection method allowed us to reach many individuals across the US and made data collection feasible during the lockdown period. However, the online nature of data collection makes it more challenging to verify participants’ responses and to ensure that they meet the appropriate inclusion criteria. A brief attention check at the beginning of the study aimed to mitigate some of the risks of inattentive or random responses. The widespread, online data collection procedures could also have resulted in selection biases. Specifically, there may have been meaningful differences between those who opted to participate in the study and those who chose not to participate. The sample was also limited in terms of racial and ethnic diversity. We could control for differences in age and gender; however, we did not have a large enough sample to examine any differences across racial or ethnic groups.

Additionally, the cross-sectional nature of this study precludes any causal conclusions. Due to the inclusion of unique respondents at each time point, we cannot determine whether differences observed across the data collection time points were more attributable to individual characteristics of each sample than to the progression of the COVID-19 pandemic. To mitigate the impact of this limitation, we controlled for several demographic variables that differed between the groups at different time points. Of note, the observed changes in boredom remained significant when controlling for these demographic differences. This finding suggests that the changes in these variables are not solely the result of between-group differences. It is possible that we would have observed similar increases in boredom across time in non-pandemic conditions. However, the findings of this study are consistent with longitudinal reports from other parts of the world showing increases in boredom over the pandemic, e.g., [2]. Thus, although the study design inherently limits the conclusiveness of the findings, we believe the results suggest that increases in boredom were likely, at least in part, related to the pandemic.

Although some of the relationships observed in this study are well-documented (e.g., boredom and substance use), others are more preliminary and require replication. Specifically, the differential associations between recent state boredom, boredom proneness, and perceptions of the future would be a promising area for future research. Studies in experimental or more controlled settings could provide insight into how boredom proneness and state boredom might affect the ways individuals think about their futures. This is especially relevant in the current context of widespread uncertainty about the future of the pandemic and the world as we know it.

## 5. Conclusions

In summary, this study demonstrated increases in boredom proneness and recent state boredom over the first several months of the COVID-19 pandemic. Additionally, the results demonstrated a rise in recent state boredom compared to pre-pandemic reports. Similar to recent studies from various parts of the world, boredom was associated with increased substance use, psychological distress, and a more negative view of the future. However, when examining the unique contributions of boredom proneness and state boredom, boredom proneness emerged as a correlate of a bleak outlook, greater substance use, fewer COVID-19 precautions, and worse mental health outcomes. Conversely, high levels of recent state boredom were not associated with many of these adverse outcomes but with a more hopeful outlook.

Future studies should further explore the differential impact of state boredom and boredom proneness on mental health outcomes. Moreover, the results suggest that boredom proneness is a critical vulnerability factor in the context of the COVID-19 pandemic, one that affects the individual and others in their social environments. For instance, highly boredom-prone participants took fewer COVID-19 precautions in this study. Similarly, previous studies have shown that boredom proneness is associated with less compliance with COVID-19 protocols [27,28,29] and more rule breaking [27,30]. High levels of boredom proneness deserve careful consideration when assessing psychological health in clinical settings, especially in contexts where high levels of state boredom are likely to be present. Increasing awareness and attention to the relevance of boredom may help individuals identify and develop strategies for boredom management. In turn, this may lead to better mental health outcomes for individuals who are highly boredom prone.

## Figures and Tables

**Table 1:** Demographics Means, Standard Deviation, and Frequencies.

	T1 (*N* = 200)	T2 ( *N* = 196)	T3 ( *N* = 183)	T4 ( *N* = 201)
Variable	*M* (*SD*)	*M* (*SD*)	*M* (*SD*)	*M* (*SD*)
**Age**	40.11 (13.51)	37.11 (11.501)	38.74 (11.91)	34.27 (9.02)
**Variable**	***N* (*%*)**	***N* (*%*)**	***N* (*%*)**	***N* (*%*)**
**Gender**				
Male	104 (51.7)	109 (55.3)	106 (57.6)	116 (57.7)
Female	96 (47.8)	84 (42.6)	77 (41.8)	83 (41.3)
Non-binary	-	2(1.0)	-	-
Missing Data	1 (0.5)	2 (1.0)	1 (0.5)	2 (1.0)
**Race/Ethnicity**				
White	145 (72.1)	129 (65.5)	132 (71.7)	151 (75.1)
Latinx/Hispanic	16 (8.0)	16 (8.1)	4 (2.2)	3 (1.5)
Asian	5 (2.5)	30 (15.2)	15 (8.2)	9 (4.5)
Black	20 (10.0)	12 (6.1)	18 (9.8)	16 (8.0)
Native American	11 (5.5)	9 (4.6)	9 (4.9)	22 (10.9)
Bi-Racial	4 (2.0)	1 (0.5)	6 (3.3)	-

*Note*: T1 = Time point 1; T2 = Time point 2; T3 = Time point 3; T4 = Time point 4; *M* = mean, *SD* = standard deviation.

**Table 2:** Descriptive Statistics for Boredom at Each Time Point.

Time		Min	Max	*M*	*SD*
T1	SBPS	8	56	30.49	12.44
	SBM	8	52	30.18	10.81
T2	SBPS	8	56	32.96	11.68
	SBM	8	56	32.23	9.70
T3	SBPS	8	56	31.68	11.33
	SBM	8	56	31.21	9.99
T4	SBPS	8	56	37.17	9.62
	SBM	8	56	35.07	8.68
Pre-COVID	SBPS	-	-	-	-
	SBM	8	56	26.36	9.94

*Note*: SBPS = Short Boredom Proneness Scale; SBM = State Boredom Measure; T1 = Time point 1; T2 = Time point 2; T3 = Time point 3; T4 = Time point 4; Min = minimum score reported; Max = maximum score reported; *M* = mean; *SD* = standard deviation.

**Table 3:** Correlations Between Boredom and Psychological Distress, Loneliness, Optimism, and Hope.

	1	2	3	4	5	6	7	8
1. SBM	--							
2. SBPS	0.74 **	--						
3. DASS S	0.53 **	0.69 **	--					
4. DASS A	0.51 **	0.68 **	0.88 **	--				
5. DASS D	0.52 **	0.70 **	0.89 **	0.86 **	--			
6. LS	0.34 **	0.53 **	0.56 **	0.52 **	0.63 **	--		
7. LOT-R	−0.23 **	−0.40 **	−0.42 **	−0.37 **	−0.48 **	−0.56 **	--	
8. HS	0.08 *	−0.04	−0.10 **	−0.06	−0.18 **	−0.41 **	0.47 **	--
9. FAS	0.12 **	−0.02	−0.15 **	−0.13 **	−0.23 **	−0.44 **	0.43 **	0.63 **

*Note.* ** = *p* < 0.01; * = *p* < 0.05. SBM = State Boredom Measure; SBPS = Short Boredom Proneness Scale; DASS = Depression, Anxiety, and Stress Scales; D = Depression; S = Stress; A = Anxiety; LS = UCLA Loneliness Scale; LOT-R = Revised Life Orientation Test; HS = Hope Scale; FAS = Future Appraisals Scale.

**Table 4:** Correlations Between Boredom, Substance Use, and Changes in Substance Use.

	1	2	3	4	5	6	7
1. SBM	--						
2. SBPS	0.74 **	--					
3. Alcohol use	0.32 **	0.33 **	--				
4. ∆: Alcohol	0.21 **	0.21 **	0.35 **	--			
5. Marijuana use	0.35 **	0.46 **	0.47 **	0.31 **	--		
6. ∆: Marijuana	0.18 **	0.18 **	0.28 **	0.47 **	0.39 **	--	
7. Drug use	0.42 **	0.56 **	0.48 **	0.34 **	0.70 **	0.35 **	--
8. ∆: Drug	0.20 **	0.23 **	0.28 **	0.48 **	0.43 **	0.65 **	0.41 **

*Note.* ** = *p* < 0.01; * = *p* < 0.05. SBM = State Boredom Measure; SBPS = Short Boredom Proneness Scale; ∆ = change.

## Data Availability

Data are available from the study authors upon request.
